# Ion Channels and Personalized Medicine in Gynecological Cancers

**DOI:** 10.3390/ph16060800

**Published:** 2023-05-29

**Authors:** Ana Ramírez, Ingrid Ogonaga-Borja, Brenda Acosta, Andrea Jazmín Chiliquinga, Jaime de la Garza, Patricio Gariglio, Rodolfo Ocádiz-Delgado, Cecilia Bañuelos, Javier Camacho

**Affiliations:** 1 Facultad de Ciencias Químicas e Ingeniería, Universidad Autónoma de Baja California, Calzada Universidad 14418, Parque Industrial Internacional, Tijuana 22390, Mexico; aramirez23@uabc.edu.mx; 2 Grupo de Investigación de Ciencias en Red, Universidad Técnica del Norte, Av. 17 de Julio 5-21, Ibarra 100105, Ecuador; imogonogab@utn.edu.ec (I.O.-B.); bjacostam@utn.edu.ec (B.A.); ajchiliquinga1@utn.edu.ec (A.J.C.); 3 Departamento de Farmacología, Centro de Investigación y de Estudios Avanzados del Instituto Politécnico Nacional (CINVESTAV-IPN), Av. Instituto Politécnico Nacional 2508, Ciudad de Mexico 07360, Mexico; 4 Unidad de Oncología Torácica y Laboratorio de Medicina Personalizada, Instituto Nacional de Cancerología (INCan), San Fernando No. 22, Tlalpan, Ciudad de Mexico14080, Mexico; jdelagarza1@prodigy.net.mx; 5 Departamento de Genética y Biología Molecular, Centro de Investigación y de Estudios Avanzados del Instituto Politécnico Nacional (CINVESTAV-IPN), Av. Instituto Politécnico Nacional 2508, Ciudad de Mexico 07360, Mexico; vidal@cinvestav.mx (P.G.); wilox@cinvestav.mx (R.O.-D.); 6 Programa Transdisciplinario en Desarrollo Científico y Tecnológico para la Sociedad, Centro de Investigación y de Estudios Avanzados del Instituto Politécnico Nacional (CINVESTAV-IPN), Av. Instituto Politécnico Nacional 2508, Ciudad de Mexico 07360, Mexico; cebanuelos@cinvestav.mx

**Keywords:** gynecological cancer, personalized medicine, ion channels, ovarian cancer, endometrial cancer, cervical cancer

## Abstract

Targeted therapy against cancer plays a key role in delivering safer and more efficient treatments. In the last decades, ion channels have been studied for their participation in oncogenic processes because their aberrant expression and/or function have been associated with different types of malignancies, including ovarian, cervical, and endometrial cancer. The altered expression or function of several ion channels have been associated with tumor aggressiveness, increased proliferation, migration, invasion, and metastasis of cancer cells and with poor prognosis in gynecological cancer patients. Most ion channels are integral membrane proteins easily accessible by drugs. Interestingly, a plethora of ion channel blockers have demonstrated anticancer activity. Consequently, some ion channels have been proposed as oncogenes, cancer, and prognostic biomarkers, as well as therapeutic targets in gynecological cancers. Here, we review the association of ion channels with the properties of cancer cells in these tumors, which makes them very promising candidates to be exploited in personalized medicine. The detailed analysis of the expression pattern and function of ion channels could help to improve the clinical outcomes in gynecological cancer patients.

## 1. Introduction

**Figure 1:**
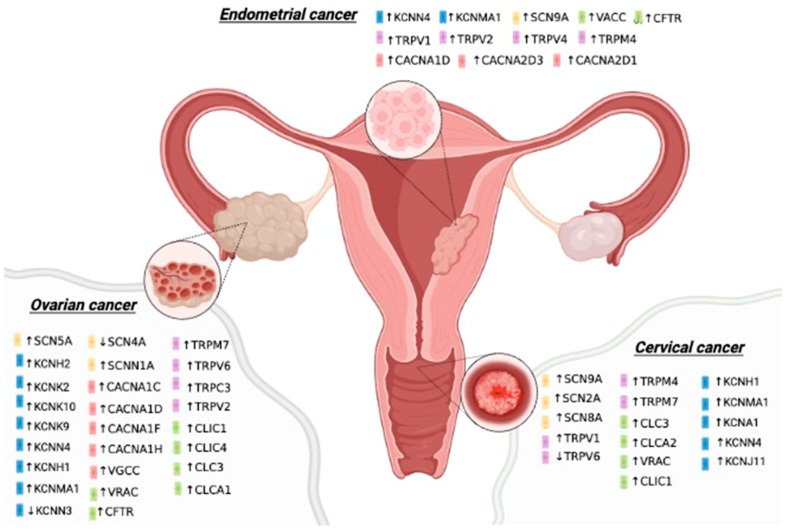
Altered expression of ion channels in gynecological cancers. A variety of ion channels are over- or under- expressed in gynecological cancer tissues (Endometrial, Cervical, Ovarian), and they are suggested to be involved in either tumor development and/or overall survival. Potassium channels are shown in blue, sodium channels in yellow, calcium channels in red, TRP channels in purple, and chloride channels in green. The increased or diminished expression of channels conducting the same type of ion in the same tissue suggest diverse roles and mechanisms of action of ion channels in cancer.

Ion channels are integral membrane proteins that allow the passage of ions through the plasma membrane and participate in diverse biological functions, from regulating the membrane potential to promoting signal transduction, contraction, and secretion, among many others. The physiological relevance of ion channels is highlighted by gene mutations or aberrant protein expression that produce a range of pathologies in humans [1], including cancer [2]. Abnormal expression of many ion channels has been identified in gynecological tumors (Figure 1) [3], and this expression has been linked to oncogenic processes. 

**Figure 2:**
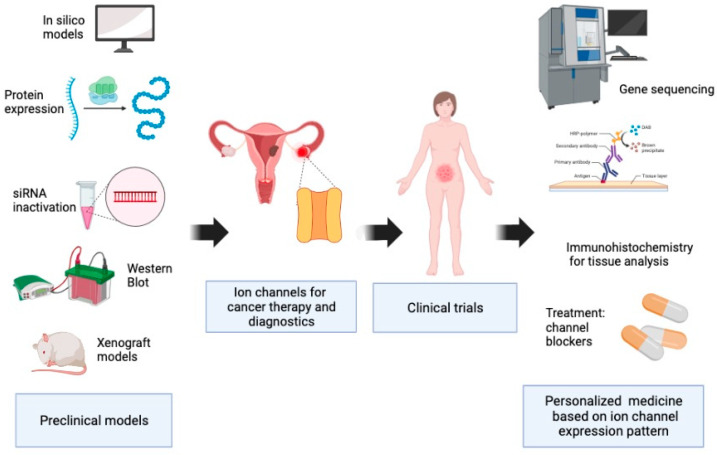
Ion channels as potential tools for personalized medicine. Ion channels have been studied for their role in gynecological cancers by different techniques and are promising candidates to be used as diagnostics biomarkers and targeted therapy, as well as prognosis and overall survival markers in gynecological cancers. The preclinical observations can be translated into the clinic investigation for specific patterns or signatures of ion channel expression.

Despite significant advances in the treatment of gynecological cancers, the five-year survival rate has not improved significantly for endometrial and ovarian cancer [4], and in some developing countries, the mortality-to-incidence ratio of cervical cancer remains high. Therefore, it is necessary to find effective biomarkers and consider the role that ion channels play in the development of gynecological cancers. In this review, we describe the association of ion channels with tumor cell properties, clinical–pathological characteristics, and outcomes in ovarian, cervical, and endometrial cancers. Besides, we discuss the potential use of ion channels in personalized medicine, which may benefit gynecological cancer patients (Figure 2). 

First, we will focus on some of the most studied ion channels in gynecological cancers, namely, the family of potassium channels. Then, we will move into the analysis of sodium, calcium, and chloride channels.

## 2. Potassium Channels

The potassium channel family is the most widely distributed group of ion channels, composed of dimers or tetrameric integral membrane proteins that regulate the flux of potassium ions. They are divided into four families based on the classification of the International Union of Basic and Clinical Pharmacology (IUPHAR): (i) voltage-gated K^+^ channels (K_v_) encoded by forty genes in twelve subfamilies, (ii) inwardly rectifying K^+^ channels, (K_ir_) encoded by fifteen genes classified in seven subfamilies, (iii) calcium- and sodium-activated potassium channels (K_Ca_, K_Na_) encoded by eight genes in five subfamilies, and (iv) two-pore domain K^+^ channels (K_2P_) encoded by fifteen genes in six subfamilies [5]. Potassium channels are aberrantly expressed in different cancer cell lines and cancer tissues [6], and there is mounting evidence that supports the association of potassium channels with the hallmarks of cancer, including cell proliferation, invasion, and migration; in accordance, blocking or suppressing their expression or activity has antineoplastic features in different types of tumors in both, in vitro and in vivo studies, strongly suggesting them as candidates for targeted therapy [7]. 

Next, we will go into details regarding the association between K^+^ channels and gynecological cancers (Table 1).

### 2.1. Endometrial Cancer

In endometrial cancer (EC), overexpression of potassium channels has been reported in several cell lines and tumor tissues [8]. The intermediated-conductance Ca^2+^-activated K^+^ channel 3.1. (K_Ca_3.1, KCNN4) mRNA expression and the corresponding protein level (detected by Western blot) are increased in EC tissues, compared to normal tissue and atypical hyperplasia. Inhibition of KCNN4 activity by blocking the channel with clotrimazole or TRAM-34, or knockout with siRNA, reduces HEC1-A and KLE cell proliferation by arresting the cells in the G0/G1 phase [9], and this was associated with reduced expression of cyclin D1 and cyclin E1 in HEC1-A and Ishikawa cells [10]. On the contrary, activation of KCNN4 induces cell cycle progression to G1 phase [9]. Furthermore, treatment of HEC1-A and Ishikawa cells with TRAM-34 reduces migration with a reduction in MMP-2 expression [10]. In nude mice with tumors of HEC1-A cells that are treated with TRAM-34 or clotrimazole, results showed smaller tumor development versus control groups [9]. Other potassium channel blockers, such as glibenclamide (GLI), 4-aminopyridine (4-AP), and tetraethylammonium (TEA), suppress cell proliferation, besides GLI and 4-AP also reduce migration in the HEC1-A cell line [11]. 

Another K^+^ channel, the large-conductance voltage and Ca^2+^-activated K^+^ channel (K_Ca_1.1, KCNMA1), is involved in cancer cell proliferation and migration, and it is aberrantly expressed in many types of tumors, including cervical [12], ovarian [13], and endometrial cancers [14,15]. It is involved in cancer cell proliferation and migration. Overexpression of KCNMA1 in HEC-1-B cells by transient transfection increases viability, promotes the cell cycle to S and G2/M phases, and favors migration. Furthermore, the contrary effect is obtained with the inhibitor IBTX or knockdown of KCNMA1. These treatments reduce proliferation and migration, and in vivo studies show a diminished tumor growth rate in nude mice with HEC-1-B xenografts [15]. In 185 tissue samples of type I endometrial cancer, protein expression of KCNMA1 by immunohistochemistry was compared with 40 normal endometrium tissues and 38 atypical endometrial hyperplasia tissues; a significantly higher expression was found in all layers of type I endometrial cancer, and there was more correlation with higher FIGO (International Federation of Gynecology and Obstetrics) stages and lymph node metastasis samples, suggesting the association of these channels with poor prognosis [14]. Thus, despite the need of supplementary research to validate prognosis features and experimental research to underline the mechanism by which KCNMA1 is involved in EC, it may be a future candidate for personalized medicine schemes in patients displaying aberrant expression of the channel.

Potassium channels may have a tumor promoting role in EC, as it occurs in other malignancies. Thus, the fine regulation of these channels by EC-associated factors, including hormones, also deserves further investigation.

### 2.2. Ovarian Cancer

Potassium channels have been associated with ovarian cancer (OC) progression and prognosis. One of the most studied K+ channels in cancer, the ether-à-go-go-related potassium channel 1 (K_V_11.1, KCNH2), is widely overexpressed in many types of cancer and promotes proliferation, migration, and invasion [16]. In OC tumor tissues, higher KCNH2 mRNA and protein expression were found when compared with adjacent non-tumor tissues, and the higher expression of KCNH2 has been associated with lymph node metastasis (LNM) and distant metastasis [17]. One study of the DNA methylation profile of clear cell OC tumors found hypermethylation and reduced mRNA expression of KCNH2; the low levels of KCNH2 in clear cell OC was associated with good prognosis, emphasizing the tumor-promoting role of these channels [18]. To the contrary, microarray analysis of the potassium calcium-activated channel subfamily N member 3 (K_Ca_2.3, KCNN3) revealed a significantly lower expression in OC tissues and in drug-resistant OC, compared with normal tissue and sensitive tissues, respectively. Furthermore, lower staining of KCNN3 by immunohistochemistry was observed in 33 of 57 tissues of OC, compared to a higher expression observed in 6 controls, and a 75% lower expression in drug-resistant tissues compared to 37% in drug-sensitive tissues was observed. These latter findings were correlated with shorter disease-free survival in samples with lower KCNN3 expression, suggesting that KCNN3 could be a tumor suppressor, therapeutic target, and a marker of poor prognosis in OC [19].

The pattern expression of potassium channels in OC may reveal the potential role of these channels as tumor promoters or suppressors. The specific therapeutic approach inhibiting or inducing the expression of certain potassium channels may give advantages for each patient in personalized medicine. 

### 2.3. Cervical Cancer

Several potassium ion channels have been implicated in the progression of cervical cancer (CCa). For example, K_V_10.1 (KCNH1) is associated with proliferation, malignant transformation, and tumor growth, and various studies have been conducted to study inhibitors of KCNH1, such as tetrandrine (30 mg/kg/day), that reduce CCa tumor growth in vivo [20]. Likewise, blockage of the K_V_3.4 (KCNC4) channel with BDS-II inhibits the migration of HeLa cells by 46%, reduces the expression of vimentin, inactivates the AKT pathway by diminishing phospho-AKT (Ser473), and increases activation of PTEN, which is a negative regulator of the AKT pathway and a tumor suppressor [21]. Detecting early stages of cancer development is one of the key factors for a successful treatment. In the case of KCNMA1 (K_Ca_1.1), it has been proposed as an early marker for CCa since higher immunostaining of the protein is more prominent in high-grade dysplasia and cervical cancer tissues [12]. Western blot analysis was used to determine the voltage-gated potassium channel K_V_1.1 (KCNA1) protein expression, showing its overexpression in CCa cell lines (HeLa, SiHa, and C-33A) compared to normal epithelia tissue, and its knockdown reduces the migration, proliferation, and invasion of HeLa cells associated with decreased Hhg and Wnt protein levels and mitochondrial capacity. Besides, in 20 CCa tissue samples from patients, increased expression of K_V_1.1 was correlated with poor prognosis and reduced survival time, which suggest K_V_1.1 as a potential biomarker for tumor development and survival rate in cervical cancer [22]. To improve chemotherapy outcomes, a widely used alternative is to sensitize cancer cells to cytotoxic drugs by increasing their permeability, considering that several anticancer drugs, such as cisplatin and doxorubicin, have low membrane permeability. Bukhari et al. found that the activation of the intermediate conductance Ca^2+^-activated K^+^ channels (K_Ca_3.1), which is upregulated in CCa cells, induces H33258 uptake, a cytotoxic DNA-binding cationic dye [23]. 

The use of specific and general inhibitors of potassium channels is a very promising approach to improve CCa therapy. While some inhibitors may be used as to decrease the malignant properties of cancer cells, the overexpression of some ion channels may also be used to facilitate the entry of anticancer drugs. In this type of cancer, the precise regulation of these channels by human papilloma virus (HPV) oncogenes and hormones is also warranted.

The differential expression of potassium channels in gynecological cancers strongly suggests that different molecular mechanisms are involved in favoring tumor development. Some potassium channels are overexpressed, while others are downregulated, strongly suggesting that potassium currents or membrane potential are not the sole mechanism involved. The potential association of potassium channels with other proteins deserves further investigation.

**Table 1:** Preclinical and clinical evidence associating potassium channels with gynecological cancers.

Channel	*In Vitro*	Animal Models	Clinical Observations	Reference
Endometrial Cancer				
K_V_11.1	High expression of KCNE and HERG genes in the AN3-CA, KLE, and Ishikawa cell lines.		Higher frequency of RNA and protein expression in primary human tumors compared to non-cancerous tissues.	[24,25]
K_Ca_1.1	Silencing with siRNA reduced cell proliferation, cell migration, and p-MEK1/2 and p-ERK1/2 expression in Ishikawa cells. Overexpression promotes cell proliferation and migration, and blockage with IBTX reduces cell proliferation in HEC-1-B cells.	Silencing in xenografts transplanted in nude mice produced smaller tumors compared to control mice.	Higher protein staining in type I endometrial adenocarcinoma tissue compared to normal and atypical endometrial tissues.	[14,15]
K_Ca_3.1	Downregulation by siRNA in HEC-1-A and KLE cells inhibits proliferation. Silencing by shRNA or blockage with TRAM-34 reduces cell cycle progression, and TRAM-34 diminishes migration and MMP2 expression in HEC-1-A and Ishikawa cells.	TRAM-34 and clotrimazole reduced tumor formation of HEC-1-A cells in nude mice.	Higher expression of mRNA and protein levels in endometrial cancer tissues compared to normal tissues.	[9,10]
Cervical cancer				
K_V_1.1	Knockdown suppresses cell proliferation, migration, invasion, and protein levels of Hhg and Wnt1 in HeLa cells.	Knockdown in HeLa cells generated smaller xenograft tumors and prolonged survival in nude mice.	Higher protein expression in CCa tissues correlates with poor prognosis.	[22]
K_V_3.4	Inactivation of the AKT pathway and inhibition of cell migration by blockage with BDS-II in Hela cells.			[21]
K_V_10.1	Higher expression in HeLa, SiHa, and primary cultures of cervical cancer. Imipramine and astemizole decrease channel expression and increase apoptosis in E6/E7-transfected keratinocytes. Decreased proliferation and increased apoptosis of HeLa, SiHa, CaSki, INBL, and C-33A cells with astemizole treatment. Decreased mRNA and proliferation with calcitriol treatment in the SiHa cell line.	Inhibition of tumor growth in xenograft mice with tetrandrine treatment. Increased mRNA and protein expression in CCa tissues of transgenic mice K14E7 treated with estrogens.	Higher expression in high-grade cervical lesions compared to low-grade lesions and normal tissues.	[26,27,28,29,30]
K_ir_6.2	Overexpression of mRNA in HeLa cells, and blockage with glibenclamide reduces cell viability.		Higher expression in invasive tumors compared to low or non-invasive tumors.	[31]
K_Ca_1.1		Estradiol treatment increased protein and mRNA expression in K14E7 transgenic mice with CCa.	Higher intensity of immunostaining in biopsies of carcinoma in situ.	[12]
K_Ca_3.1	Downregulation by siRNA increased apoptosis in HeLa cells. Increased uptake of dye H33258 dependent on K_Ca_3.1 is observed in cervical carcinoma cell lines (CXT) compared to nonmalignant cervical epithelial cell strains (HCX). Clotrimazole reduces mRNA expression and changes HeLa cell morphology.		mRNA and protein overexpression in cervical cancer tissues.	[23,32,33]
Ovarian cancer				
K_2p_2.1 K_2p_10.1	Curcumin increases late apoptosis and decrease proliferation in SK-OV-3 and OVCAR-3 cells.		Expression is increased in cancer samples compared to normal ovarian samples.	[34]
K_2p_9.1	Reduction in proliferation and increase in late apoptosis in SK-OV-3 and OVCAR-3 cells with methanandamide treatment.		Significant correlation of immunostaining with tumor stage in patient biopsies.	[35]
K_V_10.1 K_V_11.1	4-aminopyridine and tetraethylammonium inhibited proliferation in SK-OV-3 cells. Imipramine increases apoptosis levels and decreases proliferation in SK-OV-3 cells. Ergtoxin inhibits the proliferation of SK-OV-3 cells.		Higher expression in OC tissues compared to noncancerous tissue.	[36,37]
K_V_10.1	siRNA targeting sensitizes SK-OV-3 and TYK cells to cisplatin-induced apoptosis.		High expression compared to normal tissues.	[38]
K_V_11.1	Berberine reduces mRNA and protein levels in SK-OV-3 cells.	Berberine decreases tumor growth in xenografts compared to the control group.	High protein expression in tumor tissues compared to non-tumor tissues.	[17]
K_Ca_1.1	Correlation of miR-31 levels and resistance to cisplatin in A2780 cells.		Loss of expression is associated with cisplatin resistance.	[39]
K_Ca_2.3			Low mRNA and protein expression in samples of ovarian serous cystadenocarcinomas compared to normal ovarian tissues and correlated with shorter disease-free and overal survival.	[19]

## 3. Sodium Channels

Sodium channels can be divided into two types, voltage-gated sodium channels (Na_V_) and epithelial sodium channels (ENaC). Na_V_ are transmembrane proteins composed by a pore-forming α–subunit of six transmembrane helical segments (S1 to S6) that can be coupled with 1 or 2 β–subunits. In humans, the family is composed by nine different alpha subunits: Na_V_1.1 (SCN1A), Na_V_1.2 (SCN2A), Na_V_1.3 (SCN3A), Na_V_1.4 (SCN4A), Na_V_ 1.5 (SCN5A), Na_V_1.6 (SCN8A), Na_V_1.7 (SCN9A), Na_V_1.8 (SCN10A), and Na_V_1.9 (SCN11A) [40]. They are found in several cell types and participate in depolarizing the cell membrane, allowing the entrance of sodium into the cell and generating action potentials in excitable cells; they are also found in non-excitable cells, participating in the regulation of proliferation and migration. Therefore, alterations in their function or expression have been associated with several types of tumors, including gynecological cancers (Table 2) [41].

### 3.1. Endometrial Cancer

Sodium channels in endometrial cancer (EC) have been less studied. In EC biopsies, Na_V_1.7 is overexpressed in 75% of samples analyzed, compared to adjacent non-cancerous tissue. A higher expression was also correlated with tumor size and local lymph node metastasis (LNM) and associated with poor prognosis. The role of Nav1.7 in EC was analyzed by activating the channel with veratridine, resulting in decreased cell apoptosis and increased cell invasion, while blocking the channel with PF-05089771 generated apoptosis and decreased invasion [42]. These results strongly suggest Na_V_1.7 channels as potential EC biomarkers and targets. The clearly identified tumor-promoting role of these channels can be exploited in personalized medicine. 

### 3.2. Ovarian Cancer

Na_V_ channels are upregulated in OC cells, predominantly Na_V_1.1, Na_V_1.3, Na_V_1.4, and Na_V_1.5, compared to normal ovaries. Blocking Na_V_ channels with 30 µM of tetrodotoxin reduces migration and invasion in Caov-3 and SK-OV-3 cell lines. In patient samples with OC with LNM, Na_V_1.5 mRNA expression level is higher compared to the normal ovary and ovarian cancer without LNM. In accordance, Na_V_1.5 protein expression was found in OC with LNM but not in the normal ovary [43]. Furthermore, blocking Na_V_1.5 channels with eicosapentaenoic acid (EPA) diminishes cell migration and proliferation in the SK-OV-3 cell line in a dose-dependent manner by increasing the inactivation of the channel [44]. In cancer cells, Na_V_ α and β subunits regulate migration, invasion, and metastasis. Data analysis of SCNN1A (sodium channel epithelial 1 subunit alpha) levels in OC patients shows a higher channel expression compared to normal ovary samples, and this correlates with a poorer overall survival. Channel overexpression was also observed in SK-OV-3, HO-8910, OVCAR-3, and CoC1 cell lines by RT-qPCR using the normal ovary cell line IOSE80 as a control. Accordingly, knockout of the SCNN1A reduced cell migration and invasion in SK-OV-3 cells; these effects were explained in part by their participation in the epithelial-to-mesenchymal transition (EMT), since SCNN1A silencing promotes the expression of E-cadherin, and reduces the expression of vimentin, N-cadherin, and Snail proteins [45]. This is consistent with Nav α subunits, where they also participate by promoting metastasis characteristic in breast and prostate cancer [46]. In the case of Na_V_ β subunits, RNA sequencing analysis revealed Nav channel expression in 48 ovarian cancer cell lines, showing a reduced expression of SCN8A (Na_V_1.6) and SCN1B (sodium voltage-gated channel beta subunit 1) in cancer cells. Furthermore, microarray analysis of OC tumors from patients showed a higher overall survival (OS) in patients that express lower levels of SCN8A, and poorer OS with lower levels of SCN1B [47]. SCN1B has been linked to tumor growth, metastasis, and vascularization in a xenograft model of breast cancer [48]. These observations suggest that the analysis of Nav channel expression may lead to improved clinical management of OC patients. In addition, these observations suggest that the association of sodium channels with cancer involves different molecular mechanisms, depending on the type of sodium channel and may include non-conducting functions via Na_V_ β subunit-mediated adhesion. This differential participation supports the precise characterization of the sodium channel expression pattern in OC patients to improve personalized medicine.

### 3.3. Cervical Cancer

In HPV16- positive cervical cancer (CCa) biopsies and in cervical cancer primary cell cultures, Na_V_1.6 and Nav1.7b mRNA overexpression was found when compared to non-cancerous biopsies; although protein expression was found in CCa and non-cancerous samples by immunohistochemistry, the pattern of positive cells was more widely distributed in all the sections of squamous epithelial tissue in CCa biopsies [49]. Na_V_1.6 expression was related to increased invasive capacity in CCa cells, and protein expression was more abundant in CCa than in noncancerous biopsies [50]. On the contrary, overexpression of ENaC in CCa can be a better prognosis marker. RNA sequencing data from human CCa, showed a better outcome when SCNN1A, SCNN1B, and SCNN1G are simultaneously overexpressed; interestingly, a higher expression was observed in normal tissues and in low-grade CCa patients [51]. Therefore, ENaC channels comprise potential biomarkers for a better prognosis in CCa patients [51]. Because CCa is associated not only with HPV infection, but with other factors, including hormone use and smoking, it would be very interesting to perform preclinical studies concerning the regulation of sodium channels by CCa risk factors. In addition, epidemiological studies analyzing sodium channel expression in smokers or hormone using CCa patients is deserved.

In addition, the regulation of sodium channel expression in normal excitable cells should be studied. How are these channels regulated in such a manner that cell proliferation is controlled? Because sodium fluxes change the membrane potential, studying the regulation of membrane potential by cancer-associated factors (and vice-versa) will provide relevant insights into cancer development.

**Table 2:** Preclinical and clinical findings correlating sodium channels with gynecological cancers.

Channel	*In Vitro*	Animal Models	Clinical Observations	Reference
Endometrial Cancer				
Na_V_1.7	Blockade decreases invasion and promotes apoptosis, where activation increases invasion in primary cultures.		mRNA overexpression associated with tumor size, metastasis, and poor prognosis.	[42]
Cervical cancer				
Na_V_1.2	High mRNA expression in primary cultures transfected with the E7 oncogene.		High mRNA expression in cancerous biopsies compared to normal biopsies.	[52]
Na_V_1.6	mRNA overexpression in primary cultures increases invasive cell capacity, mediated by MMP2 activity.		More extensive protein pattern expression in tissue biopsies of cervical cancer compared to non-cancerous biopsies.	[49,50]
Na_V_1.7	mRNA overexpression in primary cultures.		More extensive protein pattern expression in tissue biopsies of cervical cancer compared to non-cancerous biopsies.	[49]
Ovarian cancer				
Na_V_1.5	Blockade with TTX decreases cell migration and invasion in Caov-3 and SK-OV-3 cells. EPA inactivates the channel and reduced migration and proliferation in SK-OV-3 cells.		Express in ovarian cancer with lymph node metastasis but not in normal ovary.	[43,53]
Na_V_1.6	RNAseq data analysis shows lower expression in 48 ovarian cell lines.		Higher overall survival in patients with lower expression.	[47]
SCNN1A	Overexpressed in SK-OV-3, HO-8910, OVCAR-3, and CoC1 cell lines.		Overexpressed in sample patients obtained by database analysis.	[45]

## 4. Calcium and TRP Channels

Calcium channels (CC) comprise a wide variety of membrane proteins that serve as the entry port of calcium into the cells or organelles, acting as a second messenger for a range of cellular pathways. CC participate in almost every biological process in the body, including proliferation, migration, apoptosis, and differentiation [54]. One family of CC has been widely studied in cancer, the voltage-gated calcium channels (VGCC), which are a group composed of three subfamilies: (i) high-voltage activated dihydropyridine-sensitive (Ca_V_1.1–1.4) channels, which mediate the L-type Ca^2+^ currents; (ii) the high- to moderate-voltage activated dihydropyridine-insensitive (Ca_V_2.1–2.3) channels, which mediate P/Q-type, N-type, and R-type Ca^2+^ currents, respectively, and (iii) the low-voltage-activated (Ca_V_3.1–3.3) channels, which mediate T-type Ca^2+^ currents [55]. 

The TRP channels (transient receptor potential channels) are non-selective cation channels that are activated by different stimuli (ligands, temperature, pH, tension, pressure, etc.), causing membrane depolarization and altering different processes, including Ca^2+^ signaling in many cases. These channels are composed of six members: TRPC, TRPM, TRPV, TRPA, TRPP, and TRPML [56], and they participate in various cellular processes, and several studies have associated TRP channels with cancer progression and growth [57]. Given the role of CC and calcium signaling in cancer, several CCs have been proposed as new anticancer therapeutic targets. In addition, the use of calcium channel blockers in cardiovascular diseases has been considered for drug repurposing as anticancer drugs. The huge diversity of CC in cellular pathways converts them into promising channels to be considered in the personalized medicine of gynecological cancers (Table 3). 

### 4.1. Endometrial Cancer

Several CCs have been described as overexpressed in EC. Biopsies of primary and metastatic endometrial cancer samples detected TRPV2 and TRPC1 mRNA expression levels, observing a higher expression in metastatic samples and a correlating mesenchymal phenotype, which is a malignant hallmark of EC; in particular, TRPV2 expression predicted disease recurrence in primary tumor biopsies, indicating that TRPV2 may be a biomarker for EC progression [58]. On the contrary, TRPM4 expression was found in low-risk endometrial tumors and correlated with the epithelial phenotype observed in less aggressive EC tumors [58]. Bioinformatic analysis from The Cancer Genome Atlas (TCGA) identified differentially expressed genes related to calcium; the results predicted a higher OS in EC patients with samples that expressed TRPM4 channels, while CACNA2D1 (gene encoding the auxiliary α2δ subunit of Ca_V_1 and Ca_V_2 [59]) was correlated with poorer OS [60]. Furthermore, CACNA2D1 is overexpressed in the EC cell lines HEC-108, KLE, Ishikawa, and HEC-06, participating in the proliferation and invasion of Ishikawa and HEC-06 cells. Additionally, RNA sequence analysis of TRPV4 from TCGA found higher expression in endometrial adenocarcinoma samples versus normal endometrial tissues. Moreover, the involvement of TRPV4 in cancer was also associated with cytoskeletal changes via the RhoA/ROCK1 pathway [61]. In silico analysis using TCGA and the Gene Expression Omnibus (GEO) database have proposed TRPM4 expression in tumors as a protective prognosis marker [62]. Actually, decreased TRPM4 expression is detected in EC tumors with advanced stage, high-grade, lymph node metastasis, and distant metastasis and depletion of TRPM4 in EC cells increases proliferation and metastasis, suggesting that this important CC could be a tumor suppressor [63]. Likewise, CACNA2D3 (calcium voltage-gated channel auxiliary subunit α_2_δ_3_) has been proposed as a tumor suppressor in EC, since it has been found downregulated in EC tissues compared to non-cancerous adjacent tissue, and overexpression of the channel in Ishikawa cells transfected in nude mice produced smaller tumor growth compared to the control group [64]. 

Several research groups have proposed calcium channel blockers to inhibit cancer progression in several cancer types. Amlodipine blocks CACNA2D1-mediated currents, inhibits proliferation, and increases apoptosis in Ishikawa cells, and in vivo treatment with amlodipine reduced tumor growth of xenografts of Ishikawa cells transplanted in Balb/c mice [60]. In EC patients, calcium serum levels are higher in patients with the more advanced stages II–IV than those with stage I, associating calcium levels with lymph node metastasis. Moreover, tumor biopsies of patients with stage II-IV have higher local calcium levels compared to stage I EC. Azelnidipine—a long-acting dihydropyridine calcium channel blocker—inhibits the proliferation of Ishikawa, Hec-1A, and AN3CA cells. To make azelnidipine more penetrant to the tumor microenvironment, a nanoparticle of azelnidipine was developed to enter the tumor cells by endocytosis. This nanoparticle was found to be more effective to generate apoptosis by augmentation of ER stress via CHOP-TRIB3 axis. In addition, the nanoparticle displayed a higher accumulation in the tumor site of EC-bearing mice, which developed smaller tumors. Furthermore, the combination of azelnidipine nanoparticles with medroxyprogesterone had a synergic effect in reducing the proliferation and promoting apoptosis in Ishikawa cells [65]. In summary, calcium channel blockers have significant potential for EC treatment.

Despite the broad participation of calcium ions in the malignant properties of cancer cells, the specific role of some CC may be used in the prognosis and treatment of EC, and repurposing some calcium channel blockers as anticancer drugs, will help to accelerate the clinical trials needed to improve EC therapy. 

### 4.2. Cervical Cancer

Alterations or disruption of calcium signaling has been found in CCa cell lines, and aberrant expression of calcium channels has been associated with cancer prognosis in patients [66]. One of the most studied channels in CCa is TRPV1, which is overexpressed in CCa tissues, compared with cervical intraepithelial neoplasia and normal epithelial tissue; in contrast, the tumor suppressor phosphatase and tension homolog (PTEN) expression is reduced in CCa, and the determination of both molecules (high TRPV1/low PTEN) has been proposed as a prognosis biomarker [67]. Other TRP channels have been found overexpressed in CCa samples, including the TRPM4 gene [68]. One mechanism by which it participates in CCa is through increased cell proliferation and promoting the β-catenin pathway. TRPM4 short hairpin RNA assays in HeLa cells showed decreased percentage of cells in the S phase of the cell cycle and reduced β-catenin expression, whereas induced expression of TRPM4 in T-REx-TRPM4 cells, a system under the control of a tetracycline-dependent promoter for TRPM4 in HEK293-derived cell line, promoted S and G2/M phases, DNA synthesis, and increased β-catenin expression. The activation of the β-catenin-Tcf/Lef pathway facilitates proliferation, invasion, and survival, implicated in cancer progression [69]. Another potential molecular biomarker and therapeutic target is TRPM7. Several studies have demonstrated the implication of TRPM7 in tumor cell proliferation, migration, and poor prognosis, and its aberrant expression in pancreatic and prostate cancer, and leukemia [70]. TRPM7 is overexpressed in CCa tissues [71] and has been correlated with different microRNAs which modulate gene expression. For instance, miR-543 is downregulated in CCa tissues and cell lines, is associated with poor prognosis and LNM; in agreement, overexpression of miR-543 reduced proliferation and invasion in CCa cell lines. Likewise, overexpression of miR-192-5p in Caski and SiHa cells reduces proliferation, promotes apoptosis, and suppresses migration; in vivo expression of miR-192-5p reduces tumor growth [72]. Bioinformatic analysis shows that TRPM7 is a target of miR-543 [73] and miR-192-5p [72]. Overexpression of TRPM7 decreases the tumor suppressive effects of miR-543 and miR-192-5p in CCa cells [72,73]. Furthermore, inhibition or decreased expression of TRPM7 increases apoptosis and reduces migration in C-33A and SiHa cells, and an in vivo study shows suppressed growth of CCa cells by upregulating miR-543 and downregulating TRPM7 expression through reducing ST7-AS1 expression (a long noncoding RNA associated with more aggressive features in CCa) [71]. TRPV6 has been found to be elevated in different types of cancer. However, in early-stage cervical squamous cell carcinoma tissues and CCa cell lines, its RNA and protein expression are downregulated; this reduction has been correlated with clinicopathologic tumor features, such as tumor growth, tumor type, tumor size, and differentiation grade, as well as shorter overall survival, identifying TRPV6 as a prognostic factor for early CCa patients [74].

CC expression and regulation in CCa offers a great opportunity to inhibit cancer cell proliferation by different mechanisms. For instance, calcium channel blockers or miRNAs targeting TRP channels could be used in combination with the standard anticancer therapy to potentiate the antineoplastic effect via different mechanisms. If this approach is based on the CC expression profile of each patient, the success of the anticancer therapy may be higher.

### 4.3. Ovarian Cancer

Calcium channels in OC have been widely studied; mainly several voltage-gated calcium channels have been related to proliferation, apoptosis, migration, and drug resistance. T-type Ca^2+^ channels (Ca_V_3.1 and Ca_V_3.2) have been found expressed in HO8910 and A2780 cells, and blocking the channels with NNC 55-0396 or mibefradil, or knockdown these channels, decreases proliferation and produces smaller tumors in an in vivo model of OC. Furthermore, OC patient tissues have increased T-type Ca^2+^ channel expression compared to non-cancerous tissue [75]. Likewise, reduced expression of Ca_V_3.1 (CACNA1G) and Ca_V_3.2 (CACNA1H) with siRNA in OC cells promotes apoptosis by reducing survivin (BIRC5) expression, an antiapoptotic protein that is responsible for preserving and maintaining cell survival. This was also observed by inhibition of T-type Ca^2+^ channels with mibefradil. Furthermore, an in vivo model of peritoneal metastasis treated with mibefradil showed higher sensitivity of tumor cells to carboplatin [76]. Therefore, T-type CC could be used as target therapies for OC treatment, and different CCBs may be considered for drug repurposing. Calcium channels are also overexpressed in the A2780-SP cell line, a stem-like ovarian cancer side population cell line that exhibits cancer stem cell-like characteristics, for instance, chemoresistance. A2780-SP cells had higher expression of CACNA1D, CACNA1F, and CACACNA1H compared to A2780 cells, and knockdown of these VGCC genes (CACNA1D, CACNA1F, and CACACNA1H) reduced the expression of the stem markers OCT3/4, NANOG, SOX2, ALDH1, and CD133. Moreover, increased expression of CACNA1D, CACNA1F, and CACNA1H was correlated with poor prognosis in OC patients [77]. The pool of L-type and T- type calcium channels may be a potential approach for personalized medicine in OC. 

The different CCBs used in clinical settings, and the huge number of preclinical investigations proposing new molecules as CCBs, may be considered as a synergic approach to fight gynecological cancers.

**Table 3:** Association of TRP and calcium channel expression or activity with gynecological cancers.

Channel	*In Vitro*	Animal Models	Clinical Observations	Reference
Endometrial Cancer				
Ca_V_1.3	Increased expression with 17 β-estradiol treatment in Ishikawa cells. Blockage suppresses cell proliferation and promotes apoptosis and autophagy in HEC-1A.		High expression in atypical hyperplasia and endometrial carcinoma tissues, but low in benign endometrial tissues.	[78,79]
TRPV2			Overexpressed in metastatic biopsies and correlated with high-risk tumors.	[80]
TRPV1	CBD triggers apoptosis in Ishikawa cells induced by channel activation.			[81]
TRPV4	High levels of expression in Ishikawa cells are linked to migration.	Reduced metastatic peritoneal nodules in the shTRPV4 mice group.	Bioinformatic analysis found higher expression in of EC tissues.	[61]
TRPM4	Silencing in AN3CA promoted proliferation, cell cycle, and migration in AN3CA.		Correlates with low-risk tumors in biopsies of EC and suggested as a protective gene (higher OS found by in silico analysis of RNA sequencing).	[58,62,63]
CACNA2D1	Overexpression in HEC-108, KLE, Ishikawa, and HEC-06 cells. Silencing with siRNA inhibited migration and proliferation of Ishikawa and HEC-108 cells.	Amlodipine treatment inhibits tumor growth in Balb/c mice.	In silico prognosis model of RNA sequencing found lower OS in samples overexpressing CACNA2D1, SLC8A1, and CCL2.	[60]
CACNA2D3	Overexpression inhibits cell proliferation and migration and increases apoptosis in Ishikawa cells.	Overexpression reduces tumor growth in BALB/c nude mice.	Expression is downregulated in EC tissues.	[64]
Cervical cancer				
TRPV1	Increased cell viability and colony formation.		Higher expression in CCa tissues.	[67]
TRPV6			Reduced expression is associated with poor prognosis in early-stage cervical squamous cell carcinoma.	[74]
TRPM4			Overexpression in gene sequence analysis in CCa specimens.	[68]
TRPM7	Silencing increases apoptosis and reduces migration in C-33A and SiHa cells.		Higher expression in CCa tissues.	[71]
Ovarian cancer				
Ca_V_1.2, Ca_V_1.3, and Ca_V_1.4	Calcium channel blockers increase apoptosis in tumor stem cells.	Combination of manidipine and paclitaxel inhibits tumor growth in ovarian-CSC xenograft mouse models.	High expression of CACNA1D, CACNA1F, and CACNA1H is associated with low survival rates.	[77]
Ca_V_1.2			In silico: lower expression in tissues from patients with ovarian cancer compared to normal tissues.	[82]
Ca_V_3.1 and Ca_V_3.2	Blockage with mibefradil and NNC 55-0396 reduces cell proliferation in HO8910 and A2780 cell lines. mRNA expression in A2780, A2780Cis, and IGROV-1	NNC 55-0396 slows down the formation of tumors in nude mice. Mibefradil sensitizes tumors to carboplatin in a mouse model.	Increased expression in tissues of patients with ovarian cancer.	[75,76]
VGCC and K_Ca_1.1	Inhibition with trimebutine decreases stem cell properties in A2780-SP.	Trimebutine treatment reduces tumor growth in A2780-SP xenograft mice.		[83]
T -Type and L-type calcium channels	Combination treatment of poziotinib and manidipine induces apoptosis in ovarian cancer stem cells in A2780-SP cells.			[84]
TRPC3	Decreased expression by SKF96365 reduces SK-OV-3 proliferation.	Downregulation suppressed tumor development in nude mice.	High expression in ovarian epithelial tumors.	[85]
TRPV6		SOR-C13 reduces tumor growth in SK-OV-3 xenografts.	High expression of mRNA and protein in ovarian cancer over normal tissue. SOR-C13 provides an antiproliferative mechanism through the inhibition of the channel activity.	[86,87]
TRPM7	Silencing inhibits migration and invasion in SK-OV-3 and OVCAR-3 cells.	Silencing prolongs the survival of mice bearing ovarian tumors.	Upregulated expression is associated with the EMT process.	[88]

## 5. Chloride Channels

The family of chloride channels includes the voltage-dependent ClC subfamily, the cystic fibrosis transmembrane conductance regulator (CFTR), the calcium-activated chloride channels (CLCA), maxi chloride channels, and volume-regulated chloride channels [89]. Chloride channels are involved in diverse cellular processes, including volume regulation, excitability, acidification of internal and extracellular compartments, cell cycle, and apoptosis. In gynecological cancers, some types of chloride channels are particularly overexpressed and provide tumorigenic characteristics, promoting proliferation and migration (Table 4) [90]. 

### 5.1. Endometrial Cancer

In EC, few studies have shown the participation of chloride channels in the development of carcinogenesis. CFTR is upregulated in EC tissues compared to normal endometrium, but inhibiting the expression of CFTR with specificity inhibitor CFTR (inh)-172 enhanced the proliferation and migration of the Ishikawa cell line [91]. The authors suggest that CFTR may have a tumor suppressor role. However, the regulatory mechanisms involving CFTR in EC may be opposite in vitro compared to the in vivo conditions. Additionally, in Ishikawa cells, blockade of volume-activated chloride channels (VACC) with 5-nitro-2-(3-phenylpropyl-amino)-benzoate (NPPB) inhibited invasion and regulatory volume decrease in a dose-dependent manner [92]. Voltage-dependent anion channel-1 (VDAC1) and VDAC3 are overexpressed in EC in comparison to normal tissues, and higher expression of all VDAC or protein expression of VDAC2 was associated with poor prognosis [93]. Because of the relevant role of chloride channels in normal tissue homeostasis, the study of their precise regulation, preventing tumor progression in normal conditions, should provide important hints to treat EC. 

### 5.2. Cervical Cancer

Histone and non-histone protein acetylation is a process involved in several diseases, including cancer. The acetylated intracellular chloride channel 1 (CLIC1) protein is mainly located in the nucleus and its expression is much higher in HPV-positive cervical cancer patients compared to HPV-negative and is related to a worse prognosis of the disease. Additionally, the elimination or blockage of CLIC1 decreases tumorigenesis. Interestingly, channel acetylation is increased in cancerous samples compared to normal and, therefore, it can be considered a therapeutic target [94]. In silico analysis of CLCA2 channel expression revealed a low expression in cervical cancer tissues. Positive regulation of CLCA2 has been linked to several signaling pathways, such as activation of the p53 pathway (inhibiting metastasis), positive regulation of the NOTCH pathway (decreasing tumor growth), and activity of the protease degradation pathway (involved in cell cycle regulation). CLCA2 down-regulated expression may be a molecular biomarker in the prognosis of patients with cervical squamous cell carcinoma and considered for CCa personalized medicine [95]. Without doubt, the epigenetic regulation of chloride channels represents an alternative and auxiliary approach in CCa treatment. 

### 5.3. Ovarian Cancer

Analysis of serum samples from patients with or without OC determined that the chloride channels CLIC1, CLIC4, as well as members of the tropomyosin (TPM) family, are significantly elevated in OC patients compared to normal samples. Thus, they may constitute a new option of specific biomarkers [96]. Moreover, the evaluation of the intracellular chloride channel 1 (CLIC1) by quantitative proteomic, Western blot, and immunohistochemistry assays identified a higher expression of CLIC1 in OC tissues compared to normal samples; furthermore, CLIC1 silencing in human OC cell lines made the cells more sensitive to redox stimulation and cisplatin [97]. Likewise, CLIC1 is highly expressed in tissues of patients with intraperitoneal metastasis compared to its low expression in normal tissue and OC without metastasis. In summary, CLIC-1 is a potential biomarker for the diagnosis of intraperitoneal metastasis in patients with serous epithelial OC [98], and considering CLIC1 as an OC therapeutic target is strongly suggested.

In another study, CLIC1 and CLIC4 presented a high percentage of immunostaining in malignant tumors, and CLIC4 was also found in benign tumors and limited to the nucleus; it was also determined that the high expression of CLIC4 is related to a negative survival prognosis. Meanwhile, in cell lines, colony formation, and wound healing, assays demonstrated that deletion of CLIC4 decreases cell proliferation and migration of epithelial OC cells [99]. CLIC4 channel high expression was found in the epithelium and cell stroma of OC tissues, suggesting that this channel promotes trans-differentiation to myofibroblasts. Thus, blocking CLIC4 channels is proposed as an alternative in the prevention of tumor progression by inhibiting the differentiation of fibroblasts to myofibroblasts [100].

In OC cell line A2780, the voltage-dependent chloride channel CLC3 is up-regulated [101]. Antisense oligonucleotides inhibiting CLC3 expression lowered the number of migrating cells and reduced the production of MMP and VEGF proteins, which are related to cell invasion, highlighting the potential use of CLC3 as a therapeutic target [102]. Additionally, the CLC3 channel has been investigated in OC for its role in resistance to paclitaxel (PTX). Resistant A2780/PTX cells had high mRNA expression of MDR and overexpression of the CLC3 channel. Furthermore, modulation of β-tubulin by CLC3 was identified to generate PTX resistance. Interestingly, the inhibition of CLC3 restored the cell sensitivity to PTX, making this approach a potential antitumor treatment in PTX-resistant patients [101].

The diverse cellular functions of chloride channels make clear the varied mechanisms participating in the malignant properties of cancer cells. Then, analyzing how each of these mechanisms affect cell proliferation, apoptosis, and invasion may provide not only specific anticancer treatments based on chloride channel expression, but also more general therapies targeting major cellular functions.

**Table 4:** Preclinical and clinical evidence associating chloride channels with gynecological cancers.

Channel	*In Vitro*	Animal Models	Clinical Observations	Reference
Endometrial Cancer				
VACC	Reduction of invasive endometrial cancer cells in the presence of a blocker in Ishikawa cells.			[92]
CFTR	Increased expression in Ishikawa cells.		mRNA upregulated expression in cancerous tissue.	[91]
Cervical cancer				
CLIC1	Increased mRNA levels expression in HeLa, SiHa, C-33A, and CaSki cells compared to normal human cervical epithelial cells.	Increased mRNA expression in mouse xenograft models.	Increased mRNA expression in cancerous tissue compared to normal samples.	[94]
CLC3	Associated with migration and invasion of SiHa cells through the PI3K/Akt/mTOR pathway.		Increased mRNA and protein expression in cervical squamous carcinoma compared to para-carcinoma and normal tissue.	[103]
CLCA2			Decreased expression in cervical squamous cell carcinoma tissues compared to normal tissue.	[95]
VRAC	Channel blockage reduces the proliferation of SiHa cells.			[104]
Ovarian cancer				
CLIC1	Gene silencing downregulates proliferation in A2780 cells.	Present in serum of xenograft mice model. Pulsed dendritic cells MtHsp70-CLIC1 improve the antitumor immune response in NOG mice.	High concentration in the serum of patients with ovarian cancer. High expression in ovarian cancer with peritoneal metastasis and contributes to tumorigenesis.	[96,97,98,105]
CLC3	Overexpression in paclitaxel-resistant A2780 cells Channel antisense inhibits cell proliferation in SK-OV-3 cells.			[101,102]
CLIC4			Present in the serum of OC patients. High expression in cancer tissue by immunohistochemistry.	[96,99]
CFTR	Gene knockdown inhibits cell motility and invasion in SK-OV-3 and A2780 cells.	Silencing inhibits xenograft tumor formation in nude mice.	Higher expression in ovarian cancer samples than in benign tumors and normal ovaries.	[106]

**Figure 3:**
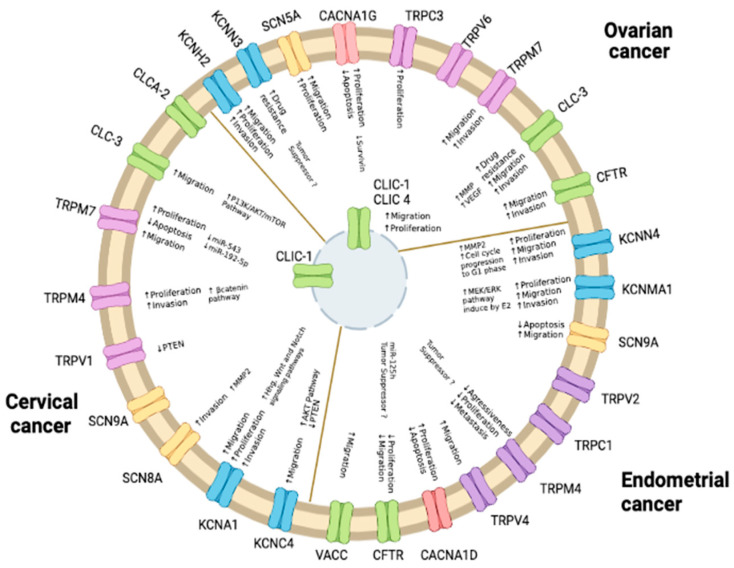
Participation of ion channels in gynecological cancers. The processes promoting tumor growth and cancer progression, involving specific ion channels, are indicated. Proven pathways involved are mentioned.

In summary, ion channels display altered expression patterns in gynecological cancers, and increasing evidence strongly suggests that they play an important role in promoting cancer growth and metastasis (Figure 3). It will be very important to establish the precise molecular mechanisms by which ion channels are altered in cancer cells, as well as to reveal the molecular mechanism by which ion channels participate in cancer development. 

## 6. Conclusions

Ion channel expression and/or activity are closely associated with clinical outcomes in gynecological cancer patients. The participation of ion channels in gynecological cancers involves tumor progression, acquisition of malignant properties, and invasive characteristics, as well as drug resistance, among other features.

Many questions remain to be solved. However, future research will help to elucidate the precise molecular mechanisms associating ion channels with gynecological cancers. Importantly, studying the regulation of ion channels in the normal tissues where they are expressed may reveal cellular components inhibiting tumor development. The study of the cellular consequences of the altered electrical regulation in cancer cells [107], as well as research concerning the fine regulation of cancer-associated channels in normal tissues and its non-conducting functions [108], is urgently necessary. 

The investigations concerning intracellular pH and cell volume regulation involving ion channels should give important insights into how normal cells control cell proliferation, despite showing high expression of ion channels dysregulated in cancer. The analysis of the potential interaction of ion channels with the tumor microenvironment deserves further investigations. 

One of the most attractive features of the role of ion channels in cancer is that several drugs targeting ion channels are already used in the clinic to treat other diseases and may be easily repurposed for cancer therapy. Because the recommended dosage and side effects of such drugs are already known, this may help to save time in clinical trials. Drug repurposing, including calcium channel blockers or antidiabetic drugs used in combination with the current standard anticancer therapy, may benefit the overall survival for gynecological cancer patients in a short time. Besides, fighting cancer cells via different mechanisms with several drugs may help to decrease the occurrence of drug resistance. If this approach is used in a personalized manner based on the expression of ion channels, the clinical outcomes of the patients may be improved. However, the potential side-effects of the drugs intended to be repurposed should be also evaluated in a personalized manner.

Countless research explaining in detail the association of ion channels with gynecological cancers is needed. However, encouraging findings point out advantages from ion channel-based therapies for effective treatment of gynecological cancers. Thus, clinical trials are warranted that may reinforce the next-generation cancer therapies [109]. Current knowledge on ion channels suggests that their application in personalized medicine schemes may improve clinical outcomes in gynecological cancer patients.

## Data Availability

Data are contained within the article.
